# Slt2 Is Required to Activate ER-Stress-Protective Mechanisms through TORC1 Inhibition and Hexosamine Pathway Activation

**DOI:** 10.3390/jof8020092

**Published:** 2022-01-18

**Authors:** Isabel E. Sánchez-Adriá, Gemma Sanmartín, Jose A. Prieto, Francisco Estruch, Francisca Randez-Gil

**Affiliations:** 1 Department of Biotechnology, Instituto de Agroquímica y Tecnología de los Alimentos, Consejo Superior de Investigaciones Científicas, Avda. Agustín Escardino 7, 46980 Paterna, Valencia, Spain; i.s.adria@iata.csic.es (I.E.S.-A.); gemma.sanmartin@iata.csic.es (G.S.); prieto@iata.csic.es (J.A.P.); 2 Departament of Biochemistry and Molecular Biology, Universitat de València, Dr. Moliner 50, 46100 Burjassot, Valencia, Spain; Francisco.Estruch@uv.es

**Keywords:** *Saccharomyces cerevisiae*, CWI pathway, UPR, glucosamine, tunicamycin, N-glycosylation, autophagy

## Abstract

Slt2, the MAPK of the cell wall integrity (CWI) pathway, connects different signaling pathways and performs different functions in the protective response of *S. cerevisiae* to stress. Previous work has evidenced the relation of the CWI pathway and the unfolded protein response (UPR), a transcriptional program activated upon endoplasmic reticulum (ER) stress. However, the mechanisms of crosstalk between these pathways and the targets regulated by Slt2 under ER stress remain unclear. Here, we demonstrated that ectopic expression of *GFA1*, the gene encoding the first enzyme in the synthesis of UDP-GlcNAc by the hexosamine biosynthetic pathway (HBP) or supplementation of the growth medium with glucosamine (GlcN), increases the tolerance of *slt2* mutant cells to different ER-stress inducers. Remarkably, GlcN also alleviates the sensitivity phenotype of cells lacking *IRE1* or *HAC1*, the main actors in controlling the UPR. The exogenous addition of GlcN reduced the abundance of glycosylated proteins and triggered autophagy. We also found that TORC1, the central stress and growth controller, is inhibited by tunicamycin exposure in cells of the wild-type strain but not in those lacking Slt2. Consistent with this, the tunicamycin-induced activation of autophagy and the increased synthesis of ATP in response to ER stress were absent by knock-out of *SLT2*. Altogether, our data placed Slt2 as an essential actor of the ER stress response by regulating the HBP activity and the TORC1-dependent signaling.

## 1. Introduction

The fungal cell wall is an external rigid structure that gives shape and integrity to the cell [1]. Its composition, made mainly of polysaccharides and glycoproteins, is continuously remodeled to allow the growth and the morphological changes required during the cell cycle [2,3,4]. Substantial changes in composition and thickness also occur in response to environmental physical stress, such as osmotic and heat stresses, in order to avoid cell membrane rupture and lysis [5]. In consonance with this, the biosynthetic pathways involved in its formation are strictly regulated in response to different signaling pathways [3,6].

In *Saccharomyces cerevisiae*, the cell wall integrity (CWI) pathway is the key pathway in controlling cell wall dynamics [7]. Signals are initiated at the plasma membrane (PM) through the cell-surface sensors Wsc1-3, Mid2, and Mtl1 [8,9], and transmitted to the downstream MAPK Slt2, which activates the transcriptional response [3]. In addition, the CWI pathway is activated not only in response to cell wall damage but also by compounds or conditions not apparently related to the cell wall. Rapamycin, alkaline pH, cadmium, and genotoxic or oxidative stresses, among others, are some of the diverse stimuli described for this pathway [7]. Furthermore, Slt2 has been reported to be involved in the regulation of different targets and cellular responses [10], i.e., mitophagy and pexophagy [11] or endoplasmic reticulum (ER) inheritance [12]. All of this evidences the role of the CWI pathway and Slt2 as central players in the protective response of *S. cerevisiae* to stress. Nevertheless, we are far from having a complete view of how the CWI pathway and Slt2 connect different signaling pathways and perform functions other than those directly related to the cell wall.

Previous work has evidenced the relation of the CWI pathway and the unfolded protein response (UPR), a transcriptional program activated upon ER stress [13]. When environmental conditions or chemical agents such as tunicamycin or β-mercaptoethanol increase the load of unfolded proteins, an ER-resident sensor Ire1 (inositol-requiring protein-1) triggers the nuclear import of Hac1, a transcription factor that upregulates the transcription of genes such as ER chaperones and folding enzymes [14,15]. Nevertheless, the activity of the Ire1-Hac1 system appears to account only for a part of the ER stress response [16]. ER stress activates the CWI pathway signaling [17], which finally results in the phosphorylation of Slt2 [18]. Consistent with this, the *slt2* mutant displays ER stress hypersensitivity, and constitutive activation of the CWI pathway provides increased ER stress resistance [18]. On the other hand, *ire1* mutants as well as strains expressing misfolded proteins have defects in cell wall integrity and cell wall stress activates the UPR in a process dependent on both Ire1 and Slt2 [19]. However, activation of Slt2 by ER stress is independent of Ire1/Hac1, and the ER stress sensitivity phenotype of the *slt2* mutant is not reversed by the presence of sorbitol, a cell-wall-stabilizing agent. Finally, an *slt2 ire1* double mutant shows additive sensitivity to ER stress compared with the parental mutants [18]. Hence, Slt2 is well-known in the ER-stress-protective response in *S. cerevisiae*, but the mechanisms of crosstalk between the CWI pathway and the UPR, and the targets regulated by Slt2 under ER stress remains unclear.

Recently, the hexosamine biosynthetic pathway (HBP), a highly conserved metabolic route from bacteria to humans [20], has emerged as one of the key sensors for cellular nutrition because the synthesis of its final product, UDP-N-acetylglucosamine (UDP-GlcNAc), is critically dependent on intermediates from a number of metabolic branches, including glucose, amino acids, fatty acids, and nucleotide [21]. The first committed and rate-limiting step of the HBP is mediated by glutamine:fructose-6-phosphate amidotransferase, encoded by the yeast *GFA1* gene [22], which converts fructose-6-phosphate and glutamine into glucosamine-6-phosphate (GlcN-6-P) and glutamate. Through four enzymatic steps, the HBP provides UDP-GlcNAc, an essential amino sugar donor for glycosylation of proteins and lipids, and for the biosynthesis of chitin [23]. Transcription of *GFA1* is upregulated by CWI pathway signaling [24], which results in higher levels of chitin [25]. Quite remarkably, X-box binding protein 1 (Xbp1s), the human homolog of yeast *HAC1*, is a direct transcriptional activator of the HBP [26]. Thus, the HBP appears to connect the cell wall and ER stress signaling by regulating the UDP-GlcNAc supply. Nevertheless, the physiological relevance of this role in the phenotype of CWI-pathway and UPR mutants remains unclear.

In addition to transcriptional regulation, Gfa1 activity is regulated by UDP-GlcNAc feedback inhibition [20] and post-translational modification, two mechanisms that in human GFAT-1 appear to be coordinated [27,28]. Recently, the yeast kinase Isr1 has also been found to negatively regulate the HBP by phosphorylating Gfa1 [29]. Overexpression of *ISR1* is lethal, which is rescued by co-overexpression of *GFA1* or exogenous glucosamine, while *isr1* mutant cells display tunicamycin resistance, implying increased protein glycosylation by enhanced UDP-GlcNAc availability [29]. Consistent with this, genetic mutagenesis screening in *Caenorhabditis elegans* has identified gain-of-function mutations in GFAT-1 that suppress tunicamycin-induced ER stress [30]. Likewise, increased synthesis of UDP-GlcNAc by exogenous supplementation of HBP intermediates provided similar results [30]. Evidence of a link between HBP metabolites and cellular protein quality control, leading to improved protein homeostasis, has been also reported [30]. However, neither N-glycosylation nor UPR signaling appeared to be affected by increased UDP-GlcNAc [30]. Hence, the regulation of the flux through the HBP is key in allowing cells to face proteotoxicity, although the exact molecular mechanisms that operate under this condition need to be clarified.

Here, we have studied the ER-stress-sensitive phenotype of cells lacking Slt2 and its relationship with the activity of the HBP and the role of the MAPK in controlling the activity of Gfa1. Furthermore, the direct and indirect effects of exogenous glucosamine in promoting ER-stress resistance have also been analyzed. Overall, our work highlights the importance of Slt2 in regulating the HBP and TORC1 activity upon ER stress, which determines the load of ER-incoming proteins and the bioenergetics of the protective response.

## 2. Materials and Methods

### 2.1. Strains and Plasmids

The *S. cerevisiae* strains, oligonucleotides, and plasmids used in this study are listed in the Supplementary Materials (Tables S1–S3). The *CHS3* deletion in the *slt2* mutant strain (Table S1) was carried out by PCR-based gene replacement using the hphMX4 module in the pAG32 plasmid (Table S3) as a template and synthetic oligonucleotides (Table S2). Detection of the correct gene disruption and tagging was performed by diagnostic PCR [31], using a set of oligonucleotides (Table S2), designed to bind outside of the replaced gene sequence and within the marker module.

### 2.2. Media, Culture Conditions, and Stress Sensitivity Tests

Previously described standard methods were followed for media preparation [32]. Yeast cells were cultured at 30 °C in YPD (1% yeast extract, 2% peptone, and 2% glucose), SCD (0.67% yeast nitrogen base without amino acids (ForMedium, Hunstanton, UK), plus 2% glucose), MPD (0.17% yeast nitrogen base without amino acids and ammonium sulphate (ForMedium), 0.1% L-proline, plus 2% glucose), or SCD-Ino (0.69% yeast nitrogen base without amino acids and inositol (ForMedium), plus 2% glucose). Yeast transformants carrying the geneticin (kanMX4), nourseothricin (natMX4), or hygromycin B (hphMX4) resistant module were selected on YPD agar plates containing 200 mg/L of G-418 (Sigma; St. Louis, MI, USA), 50 mg/L of nourseothricin (clonNAT; WERNER Bioagents, Jena-Cospeda, Germany), or 300 mg/L of hygromycin B (Sigma), respectively [33,34]. *Escherichia coli* DH5α host strain was grown in Luria-Bertani (LB) medium (1% peptone, 0.5% yeast extract and 0.5% NaCl) supplemented with ampicillin (50 mg/L). All amino acids, sugars, and antibiotics were filter sterilized and added to the autoclaved medium. Solid media contained 2% agar. Yeast cells were transformed by the lithium acetate method [35].

For plate phenotype experiments, cultures were diluted to OD_600_ = 0.8 and 10-fold serial dilutions spotted (2 μL) onto YPD- or MPD-agar solid media, lacking or containing glucosamine (GlcN; Sigma; cat# G4875), calcofluor white (CFW; Sigma; cat# F3543), dithiothreitol (DTT; Serva Electrophoresis GmbH, Heidelberg, Germany; cat# 20710), or tunicamycin (Tn; Enzo Life Sciences, Farmingdale, NY, USA; cat# BML-CC104) as indicated. SDS (0.003% final concentration) was added to the culture medium when proteasome inhibitors, MG132 (Selleckchem; Houston, TX, USA; cat# S2619), bortezomib (Selleckchem; cat# S1013), and delanzomib (Selleckchem; cat# S1157) were tested. Stock solutions of tunicamycin (25 mg/mL, DMSO), cycloheximide (100 mg/mL, DMSO), CFW (10 mg/mL, water), GlcN (100 mg/mL, water), and proteasome inhibitors (100 mM, DMSO) were prepared, sampled in small volumes, and stored at −20 °C until use. For each experiment, a fresh sample was thawed and diluted to the working concentration. Unless otherwise indicated, colony growth was inspected after 2–4 days of incubation at 30 °C.

### 2.3. Microscopy and Chitin Staining

To visualize the amount of chitin, exponentially growing cells (OD_600_ = 0.5) were fixed with formaldehyde, washed with PBS, and treated with 0.06% diethanolamine as formerly described [36]. Samples (50 μL) were then incubated overnight in the dark at 4 °C with 5 μL of 1 mg/mL CFW. Finally, cells were washed five times with PBS, resuspended in immunofluorescence mounting solution, and stored at 4 °C until their visualization under a Zeiss 510 Meta Confocal microscope with a 63 × Plan-Apochromat 1.4 NA Oil DIC objective lens (Zeiss, Oberkochen, Germany). Image processing was conducted with ImageJ (http://rsb.info.nih.gov/ij/, accessed on 7 October 2021.

### 2.4. qRT-PCR

Total RNA and cDNA were prepared and quantitative RT-PCR (qPCR) experiments were carried out as described previously [37]. Briefly, qPCR was performed in a DNA Engine Peltier Thermal Cycler (Bio-Rad, Hercules, CA, USA) using the SYBR Premix Ex Taq Tli RNaseH Plus Green with ROX (Takara, Shiga, Japan) and specific oligonucleotides (Table S2). Relative quantification of gene expression was determined using the comparative Ct (threshold cycle number) method analysis [38]. Fold change values were calculated as the 2^(−ΔdCt), where dCt = Ct[Target] − Ct[Housekeeping], and ΔdCt = (ΔExperimental condition) − (ΔControl). Samples were run in triplicate and normalized to *ACT1* mRNA as a housekeeping gene. Each graph is representative of at least three independent experiments.

### 2.5. Galactosidase Assay

SCD-Ura-grown overnight seed cultures were refreshed at OD_600_ = 0.1 in YPD with or without the addition of 11.5 mM GlcN and cultivated at 30 °C. When OD_600_ reached 0.5, aliquots (15 units) were withdrawn for their analysis (control), and cultures were exposed to 2 μg/mL of tunicamycin for 90 min. Cells were centrifuged, washed with Z buffer (60 mM Na_2_HPO_4_, 40 mM NaH_2_PO_4_, 10 mM KCl, 1 mM MgSO_4_), and protein extracts were prepared and processed for galactosidase activity as previously described [39]. One galactosidase unit is defined as the amount of enzyme that is able to convert 1 nmol of the substrate o-NPG per min under the assay conditions. The given values represent the mean ± SD of three independent experiments, each conducted in triplicate.

### 2.6. Preparation of Protein Extracts and Western Blot Analysis

Proteins were extracted, separated, and analyzed by SDS-PAGE and Western blot as previously described [40]. GFP-Atg8 and Gfa1-TAP were visualized by using a monoclonal anti-GFP antibody (1:3000; Roche Diagnostics, Indianapolis, IN, USA; cat# 11814460001) and soluble peroxidase-anti-peroxidase (α-PAP) antibody (1:1000; Sigma; cat# P1291), respectively. Rabbit anti-phospho Rps6 (1:10,000; kindly provided by T. Moustafa) was used to check the activity of TORC1. Total CPY and Gas1 were probed with rabbit polyclonal anti-CPY (1:10,000; antibodies-online, Aachen, Germany; cat# ABIN607698) and anti-Gas1 (1:10,000; a gift from H. Riezman). Hac1 and Kar2 were detected with a mouse monoclonal XBP1-antibody (1:1000; Santa Cruz Biotechnology, Santa Cruz, CA, USA; cat# sc-8015) and rabbit monoclonal anti-Kar2 (1:3000; Santa Cruz Biotechnology; cat# 33630), respectively. N-glycosylated proteins were visualized with horseradish peroxidase-conjugated concanavalin A (ConA-HRP; 1:10,000; Sigma; cat# L6397). Mouse monoclonal phosphoglycerate kinase 1 (Pgk1; 1:3000; ThermoFisher, Waltham, MA, USA; cat# 459250) and rabbit glucose-6-phosphate dehydrogenase (G6Pdh) antibody (1:3000; Cell Signaling, Danvers, MA, USA; cat# 8866) were used as loading control. The secondary antibodies used were HRP-conjugated goat anti-rabbit (1:2000; Cell Signaling; cat# 7074) or rabbit anti-mouse (1:5000; Dako, Carpinteria, CA, USA; cat# P0260). Blots were carried out and images were captured as described elsewhere [41].

### 2.7. Cycloheximide Treatment

Pulse analysis of Gas1 degradation in wild-type and *slt2* mutant cells grown in YPD-lacking or containing GlcN (OD_600_ = 1.0) was carried out by adding cycloheximide (CHX) at a concentration of 100  µg/mL. Aliquots were immediately withdrawn (control), and cultures were shaken at 30 °C for an additional 60, 120, or 180 min. Samples at each time point were centrifuged, washed, and processed as described above for Western blot analysis of Gas1.

### 2.8. ATP Assay

Overnight-grown YPD seed cultures of the BY4741 wild-type and *slt2* mutant strain were refreshed at OD_600_ = 0.1 in the same medium lacking or containing GlcN and cultivated at 30 °C until OD_600_ = 0.3. Aliquots were withdrawn for their immediate analysis (control), and cultures were split 1:2 and incubated in the presence or absence of 2 μg/mL tunicamycin. At different times during growth, 100 μL samples were analyzed for ATP levels using the CellTiter-Glo^®^ Luminescent Assay following the manufacturer’s instructions (Promega, Madison, WI, USA). The ATP level in the cell suspensions was calculated after correcting for the reagent background using the signal produced by an ATP standard as reference. Values provided are expressed as nmol of ATP per OD_600_ and represent the mean (± SD) of triplicate assays. ATP kinetics for each strain and condition was repeated at least three times.

### 2.9. Statistical Analysis

Sample averages were compared using a Student’s *t*-test with Excel software (Microsoft, Redmond, WA, USA). Different letters represent significant differences at a *p* < 0.05 probability level.

## 3. Results

### 3.1. Activation of the HBP Provides Tunicamycin Tolerance and Rescues the ER-Stress-Sensitivity Phenotype of the slt2 Mutant

**Figure 1:**
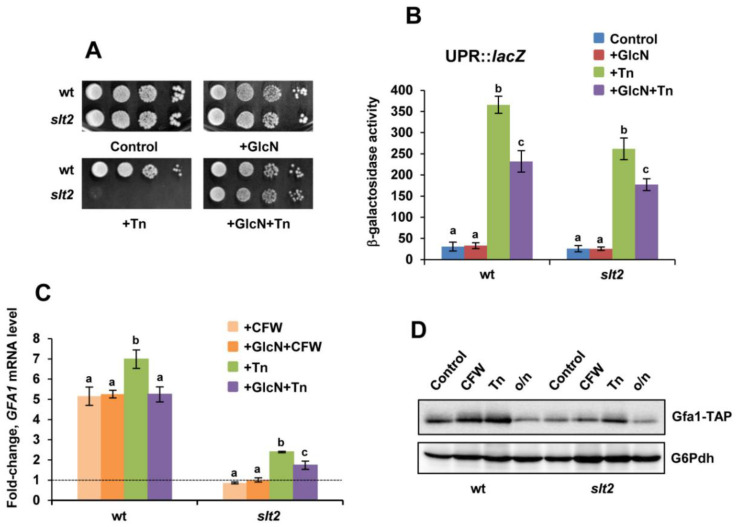
Activation of the HBP reduces the UPR response and provides ER-stress tolerance. (**A**) Serial dilutions (1–10^−3^) of YPD-grown cultures (OD_600_ ~ 0.8) of the BY4741 wild-type (wt) and its isogenic *slt2* mutant were spotted (2 μL) onto YPD plates that lacked or contained glucosamine, GlcN (11.5 mM) and/or tunicamycin, Tn (0.5 μg/mL) and were incubated at 30 °C for 2–4 days. (**B**) The activity of a UPRE::*lacZ* reporter was assayed in YPD-grown cells (OD_600_ ~ 0.5) of the indicated strains exposed to 11.5 mM GlcN and/or 2 μg/mL tunicamycin (Tn) for 90 min. Aliquots of yeast cultures were harvested and cells were assayed for β-galactosidase activity. Data represent the mean value ± SD of three independent experiments. The activity values with different letters were significantly different at *p* < 0.05. (**C**) YPD-grown cells of the indicated strains (OD_600_ ~ 0.5) were treated with 40 μg/mL calcofluor white (CFW) and/or 2 μg/mL tunicamycin (Tn) for 90 min and samples from untreated (control) and treated cultures were processed for qPCR analysis of *GFA1* mRNA. Expression differences between untreated and treated samples for the wt and *slt2* strain are shown as fold-change. Data represent the mean (±SD) of at least three independent experiments. Statistically significant (*p* < 0.05) differences are denoted with different letters. (**D**) Protein extracts from Gfa1-TAP-tagged cells of the wild-type (wt) and *slt2* mutant strain were obtained by NaOH-treatment and analyzed by regular SDS-PAGE and Western blot by using soluble peroxidase-anti-peroxidase (α-PAP) antibody as described in the Materials and Methods section. YPD cultures were grown until mid-log phase (OD_600_ ~ 0.5) at 30 °C (control) and treated with 40 μg/mL CFW or 2 μg/mL Tn for 90 min. Untreated cultures grown overnight (o/n) were also tested. The level of glucose-6-phosphate dehydrogenase (G6Pdh) was used as a loading control for crude extracts. A representative experiment is shown.

We first examined the growth of the *slt2* strain in the presence of tunicamycin, a natural inhibitor of Alg7, which induces unfolded protein stress. *ALG7* encodes the first enzyme in the N-linked glycosylation pathway [42]. As expected from previous reports [18], deletion of *SLT2* in the BY4741 wild-type strain resulted in strong sensitivity to the drug (Figure 1A). Tunicamycin induced the activation of a UPRE::*lacZ* reporter [43] both in wild-type and *slt2* mutant cells, although the activation levels were lower in the latter (Figure 1B). Similar behavior was observed when analyzing the induction of Hac1 and Kar2 by tunicamycin in wild-type and *slt2* mutant cells (Figure S1A). Kar2, a UPR-dependent ER chaperone protein [16], and Hac1, the UPR transcription factor [44,45], are well-known readouts of the UPR signaling. Finally, the overexpression of a functional mature form of *HAC1* [46] did not provide any growth advantage to *slt2* mutant cells in the presence of tunicamycin (Figure S1B).

Then, we analyzed the implication of the HBP in the phenotype of the *slt2* strain. As mentioned, transcription of *GFA1*, the gene encoding the first enzyme in the synthesis of UDP-GlcNAc by the HBP [22], has been reported to be regulated by several stress conditions, including cell-wall and ER stress [24]. As shown in Figure 1C, the induction of *GFA1*, both by tunicamycin and by calcofluor white (CWF), a fluorochrome that binds chitin [47], was mainly dependent on the presence of Slt2. Consistent with this, the abundance of the Gfa1 protein was lower in cells of the *slt2* mutant exposed to either CFW or tunicamycin (Figure 1D). This suggested that impaired HBP flux by Gfa1 downregulation might cause ER stress sensitivity accounting for the phenotype of the *slt2* strain. Indeed, exogenous addition of glucosamine, GlcN (Figure 1A), or expression of *GFA1* from a multicopy plasmid [48] rescued the ER-stress-sensitivity phenotype of the *slt2* mutant (Figure S1C). Moreover, GlcN supplementation stimulated the growth of wild-type cells in the presence of tunicamycin, indicating that the aminosugar effects are not restricted to the CWI MAPK mutant (Figure S2). Interestingly, we also note that the addition of GlcN reduced the tunicamycin-induced UPR response and the transcriptional activation of *GFA1* in both wild-type and *slt2* cells (Figure 1B,C). The aminosugar GlcN is taken up by glucose transporters and phosphorylated by *S. cerevisiae* hexokinase [49], thus increasing the synthesis of UDP-GlcNAc and bypassing the need for Gfa1 activity [50]. We conclude that the role of the CWI pathway in ER stress is largely dependent on the HBP activity.

### 3.2. The HBP Links Different Signaling Pathways in the ER Stress Response

**Figure 2:**
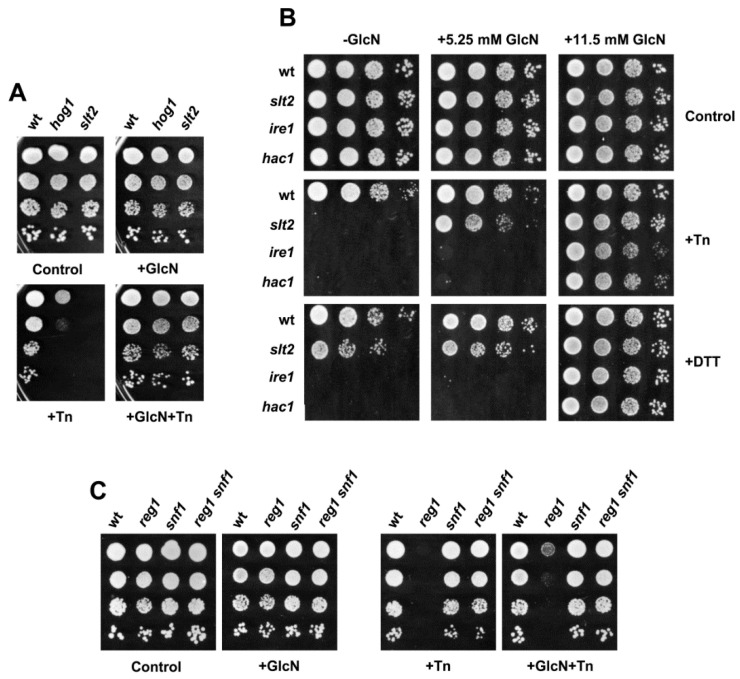
Glucosamine supplementation confers tolerance to several ER stress inducers and to mutants in different signaling pathways. (**A**) Drop test of BY4741 wild-type (wt), *hog1*, and *slt2* mutant strains. Cultures were diluted (1–10^−3^) and spotted (2 μL) onto YPD plates lacking or containing 11.5 mM glucosamine (GlcN) and/or 0.5 μg/mL tunicamycin (Tn). (**B**) Cultures of the indicated strains, BY4741 wild type (wt), *slt2*, *ire1*, and *hac1*, were assayed for growth on YPD plates lacking or containing GlcN at the indicated concentrations and/or 0.5 μg/mL Tn or 17 mM dithiothreitol (DTT). (**C**) The ER-stress tolerance of the BY4741 wild-type (wt), *reg1*, *snf1*, and *reg1 snf1* strains was inspected on YPD plates lacking or containing glucosamine (GlcN) and/or tunicamycin (Tn). Drug concentrations and cultures processing conditions were as in panel (**A**). In all cases, a representative experiment is shown.

In addition to the CWI and the UPR, other signaling pathways, among them, the osmosensing high osmotic glycerol (HOG) pathway [51], have been identified as playing a role in the protective response to ER stress [18,39]. Interestingly, the HOG and CWI pathways are positively coordinated to regulate many stress responses [6]. As shown in Figure 2A, deletion of *HOG1* caused a strong sensitivity to tunicamycin as reported [39]. However, the presence of exogenous GlcN reversed the tunicamycin sensitivity of the *hog1* strain (Figure 2A). The result led us to examine the phenotype of cells lacking *IRE1* or *HAC1*, the main actors in controlling the transcriptional and post-translational response to ER stress [15]. As expected, *ire1* and *hac1* mutant cells showed a strong sensitivity to the presence of 0.4 μg tunicamycin/mL, a phenotype that was not alleviated by the addition of GlcN at doses (5.25 mM) that provide some protection to *slt2* (Figure 2B). However, at higher GlcN concentrations (11.5 mM), *ire1* and *hac1* mutants grew as a wild-type strain (Figure 2B).

We speculated that GlcN alleviates the tunicamycin sensitivity of different yeast mutants just by increasing the synthesis of UDP-GlcNAc. Tunicamycin is structurally related to UDP-GlcNAc, and thus both could compete by binding to the active site of Alg7. However, tunicamycin inhibition has been reported to be noncompetitive in nature [52]. Consistent with this, the exogenous addition of GlcN had similar effects when dithiothreitol (DTT) was used as an ER-stress inducer (Figure 2B). The reducing agent DTT disrupts protein folding by preventing disulfide bond formation. Furthermore, GlcN was effective in partially overcoming the requirement of inositol of CWI pathway mutants lacking Bck1, the MAPKK of the CWI pathway [3], or Slt2 (Figure S3). Depletion of the membrane lipid component inositol triggers the UPR [53], likely by adversely affecting the integrity of glycosylphosphatidylinositol (GPI)-anchored proteins [53] and impairment of Ca^2+^ fluxes, thereby contributing to protein misfolding [54]. However, GlcN was unable to rescue the inositol auxotrophy of *PHO85* mutations (data not shown), a well-known regulator of sphingolipid biosynthesis [37].

Then, we examined the effects of GlcN in the nutrient-sensing Snf1-mediated catabolite repression pathway [55]. The Snf1 kinase, the *S. cerevisiae* ortholog of AMP-activated protein kinase, AMPK [56], has been reported to be involved in the regulation of the UPR [57]. Indeed, cells lacking Reg1, a regulatory subunit of the Glc7 protein phosphatase, which causes the inappropriate activation of Snf1 [56] displayed hypersensitivity to tunicamycin ([57]; Figure 2C). On the contrary, *snf1* and *reg1 snf1* mutant cells exhibited only a weak growth defect, if any (Figure 2C). We also found that GlcN provided improved tunicamycin tolerance to *snf1* and *reg1 snf1*, but this effect was scarce in cells devoted to a functional Reg1 protein (Figure 2C). Overall, the results suggest that the HBP mediates a specific protective response.

### 3.3. The Synthesis of Chitin Does Not Confer Protection against ER Stress

**Figure 3:**
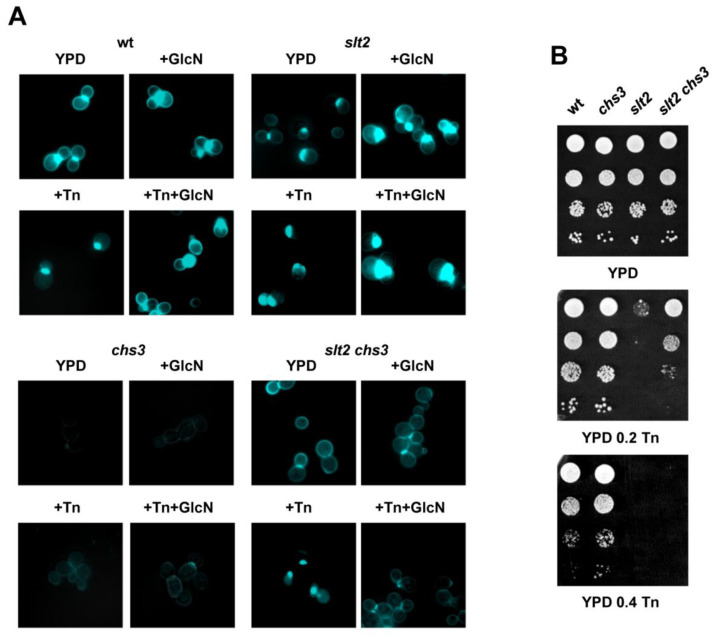
Increased synthesis of chitin in response to tunicamycin exposure is not essential for ER stress tolerance. (**A**) Exponentially growing cells of the BY4741 wild-type (wt), *slt2*, *chs3*, and *slt2 chs3* mutant strains were treated with 11.5 mM glucosamine (GlcN) and/or 2 μg/mL tunicamycin (Tn) for 90 min and processed for chitin staining with calcofluor white (CFW) and microscopy visualization as described in the Materials and Methods section. (**B**) The same strains were checked for growth in YPD medium lacking or containing tunicamycin at the indicated concentrations (μg/mL). Cultures were diluted and spotted as indicated in Figure 1. In all cases, representative experiments are shown.

The synthesis of cell wall components, particularly chitin, is a common response to stress conditions that threaten cell integrity [3]. In particular, chitin levels have been reported to increase when GlcN is added to the culture medium of yeast cells [50]. In addition, genes involved in chitin biosynthesis have been identified among those that were upregulated in tunicamycin-resistant mutants isolated by adaptive aneuploidy [58]. Consequently, we analyzed whether chitin synthesis, as measured by CFW-fluorescence microscopy, could be in part responsible for the positive effects on ER-stress sensitivity of increasing levels of intermediates of the HBP. Single *slt2*, *chs3*, and double *slt2 chs3* mutants were examined for chitin levels and tunicamycin sensitivity in the presence or absence of GlcN (Figure 3). As expected, GlcN increased the synthesis of chitin in all the strains analyzed, except in the single *chs3* mutant (Figure 3A). To our surprise, cells of the *slt2 chs3* strain exhibited enhanced chitin fluorescence as compared with the single *chs3* strain, suggesting that other chitin synthase genes were induced in the absence of Slt2. Chs3, the major chitin synthase, is responsible for more than 90% of the chitin in *S. cerevisiae* [59], but additional enzymes, Chs1 and Chs2 exist [1]. A lack of Slt2 reduced chitin levels in the lateral cell wall, but deposits still remain visible at the bud-neck region (Figure 3A), a change that was also apparent in tunicamycin-treated cells. Changes in trafficking, abundance, and subcellular location of chitin synthase enzymes could explain these results [1,50].

Elevated or reduced levels of chitin did not appear to have a great impact on the tunicamycin sensitivity of yeast cells. Although increased chitin synthesis by GlcN addition (Figure 3A) correlated with improved tolerance to tunicamycin-induced ER stress in the *slt2* mutant, the single *chs3* displayed higher tolerance than the wild type in the absence of GlcN (Figure 3B). Neither the improved synthesis of chitin in the double *slt2 chs3* mutant increased its tolerance to the drug as compared with the single *chs3* (Figure 3B). We also note that the addition of GlcN to the culture medium, either lacking or containing tunicamycin, caused a strong growth defect in *slt2 chs3* cells (data not shown). Although we have no obvious explanation for this result, it seems that the addition of GlcN may lead to energy imbalances, as UDP-GlcNAc recycling could be impaired in the context of some cell-wall mutants. We conclude that chitin synthesis is not a major determinant of ER-stress tolerance.

### 3.4. The Exogenous Addition of GlcN Reduces the Abundance of Glycosylated Proteins

**Figure 4:**
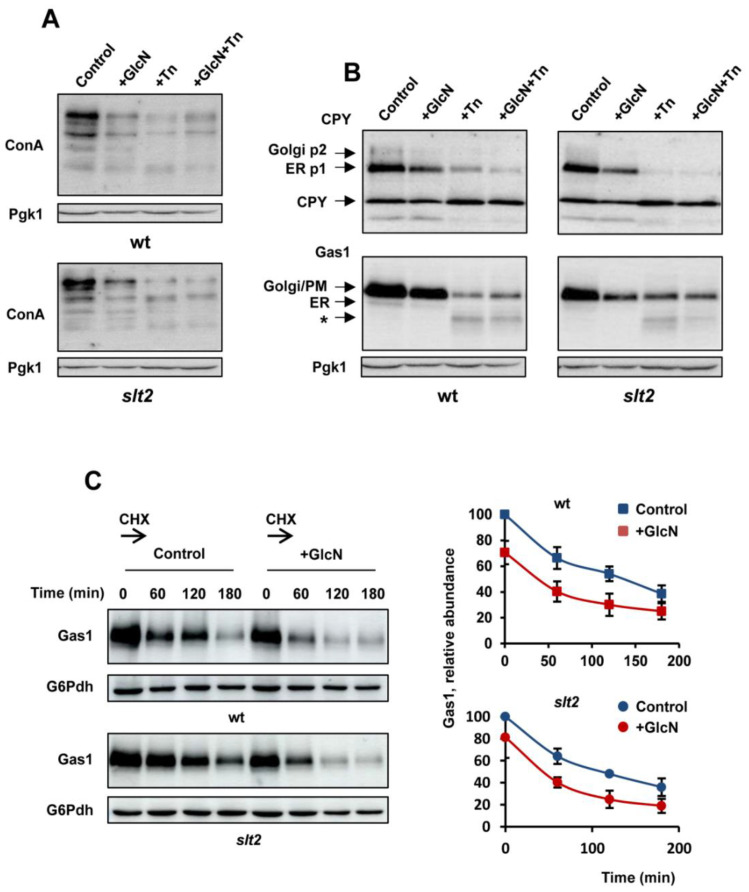
Increased HBP flux by glucosamine supplementation reduces the abundance of N-glycosylated proteins. (**A**) Protein extracts from the indicated strains, BY4741 wild type (wt) and *slt2*, were separated by SDS-PAGE and analyzed by Western blot for concanavalin A staining (ConA). Aliquots from YPD-grown cells (OD_600_ ~ 0.5) were withdrawn (control) and cultures were shaken at 30 °C for an additional 180 min in the presence of 11.5 mM glucosamine (GlcN) and/or 2 μg/mL tunicamycin (Tn). Samples at each time point were centrifuged, washed, and processed as described in Section 2. The level of phosphoglycerate kinase (Pgk1) was used as a loading control for crude extracts. A representative experiment is shown. (**B**) The indicated strains were cultivated under the same conditions and protein extracts were processed by SDS-PAGE and Western blot analysis of CPY (upper panel) and Gas1 (lower panel). The arrows show the ER-localized “p1” form of proCPY (67 KDa), the Golgy-localized “p2” form of CPY (69 KDa), and the vacuolar 61 kDa active, mature form of the enzyme. Likewise, arrows in lower panel show 105 KDa ER-form (“p1”) and the 125 KDa (“p2”) mature forms of Gas1, respectively. Bands labeled with (*) corresponded with degraded forms of Gas1. (**C**) Pulse analysis of Gas1 degradation in wild-type and *slt2* mutant cells grown in YPD-lacking or containing 11.5 mM GlcN (OD_600_ ~ 1.0) was carried out by adding cycloheximide (CHX) at a concentration of 100 µg/mL. Aliquots at the indicated times were withdrawn and protein extracts were processed by SDS-PAGE and Western blot as in panel (**B**). The level of glucose-6-phosphate dehydrogenase (G6Pdh) was used as a loading control for crude extracts. The graph shows the abundance of Gas1 at each time point relative to that of the control for GlcN-treated and -untreated samples of each strain analyzed. Data are the mean (±SD) of three independent biological replicates.

Previous work by Denzel and coworkers [30] demonstrated that increased synthesis of N-glycan precursors in the HBP improves ER protein homeostasis and extends lifespan in *C. elegans*, phenotypes that were ascribed to improved protein homeostasis, although the molecular mechanisms involved were not clarified. One possibility is that increased UDP-GlcNAc could modulate in some way N-glycosylation, the main target of tunicamycin. However, a global change in steady-state protein glycosylation in gfat-1 gain-of-function mutants of *C. elegans* has not been observed [30]. We, therefore, were interested to examine this aspect in yeast cells. Protein glycosylation as measured by N-glycan labeling with concanavalin A was recorded by Western blot in samples from wild-type and *slt2* mutant cells cultivated in the presence of GlcN and/or tunicamycin (Figure 4A). As expected, tunicamycin exposure caused a bulk reduction in N-glycosylated proteins in both wild-type and *slt2* strains. Unexpectedly, increased HBP flux by GlcN addition had a similar effect, although the reduction seemed to be less intense (Figure 4A).

Then, we checked the protein abundance and electrophoretic profile of two ER-client proteins that are often used as model secretory glycoproteins, CPY, the yeast vacuolar carboxypeptidase Y [60] and Gas1, a β-1,3-glucanosyltransferase that localizes to the cell surface via glycosylphosphatidylinositol (GPI) anchor [61,62]. As can be seen, the fraction of ER- and vacuole-localized pro-CPY forms was sharply reduced in tunicamycin-treated cells of wild-type and *slt2* cells (Figure 4B). Likewise, the abundance of Gas1 decreased in cells exposed to the drug, with the appearance of degraded forms of higher electrophoretic mobility. More importantly, GlcN exposure caused again similar effects, although the combined exposure to both tunicamycin and GlcN did not appear to result in a further reduction of protein abundance (Figure 4B). Consistent with this, a gradual loss of Gas1 abundance was observed when control cells were exposed to a pulse of GlcN (Figure S4). We also noted that decreased protein abundance by tunicamycin or GlcN exposure did not appear to affect glycolytic enzymes, such as phosphoglycerate kinase (Pgk1, Figure 4B), glucose-6-phosphate dehydrogenase (G6Pdh, Figure S4), or hexokinase PII, Hxk2 (data not shown), used as loading controls.

Finally, we wonder whether the GlcN-induced loss of Gas1 abundance reflects enhanced protein degradation. To examine this, cycloheximide (CHX) was added to wild-type and *slt2* YPD cultures lacking (control) or containing GlcN. Aliquots of cells were collected immediately and at specific time points, and protein samples were analyzed by Western blot for Gas1 relative abundance. As shown in Figure 4C, the apparent rate of CHX-induced disappearance of Gas1 was similar in GlcN-treated and -untreated cultures of either wild-type or *slt2*.

### 3.5. TORC1 Remains Active in Tunicamycin-Exposed Cells of the Slt2 Mutant Strain

**Figure 5:**
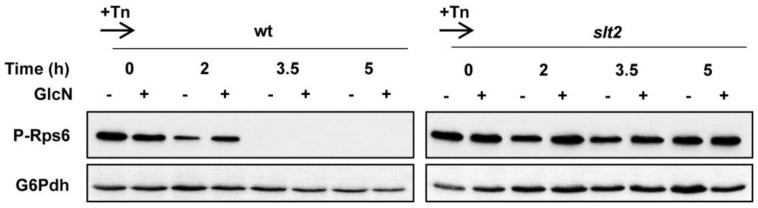
TORC1 activity is not inhibited by tunicamycin in absence of Slt2. Analysis of TORC1 activity as measured by phosphorylation of the 40S ribosomal protein S6. Protein extracts obtained from YPD-grown cultures (OD_600_ ~ 0.5) were treated with 2 μg/mL tunicamycin (Tn) for the indicated times in the presence (+) or absence (−) of 11.5 mM glucosamine (GlcN), separated by SDS-PAGE and analyzed by Western blot for phospho-Rps6 (P-Rps6). The level of glucose-6-phosphate dehydrogenase (G6Pdh) was used as a loading control for crude extracts. A representative experiment is shown.

TORC1 integrates multiple signaling pathways and plays a key role as a central stress and growth controller [63]. TORC1 activity promotes the cellular translation capacity and restricts the abundance of the proteolytic machinery [64,65], and thus, regulation of TORC1 is crucial to ensure protein homeostasis under stress conditions. Quite remarkably, tunicamycin has been reported to inhibit TORC1 signaling [66,67], and to increase—via Slt2—proteasome abundance [68]. Therefore, we first examined whether increased levels of HBP intermediates regulate TORC1 signaling. As expected, tunicamycin quickly inhibited the TORC1 activity, as measured by phosphorylation of the 40S ribosomal protein S6 (Rps6), in cells of the wild-type strain, an effect that was not mainly affected by the simultaneous addition of GlcN (Figure 5). The phosphorylation of Rps6 is a well-established readout of TORC1-dependent signaling [69,70]. To our surprise, TORC1 activity was insensitive to tunicamycin in the *slt2* mutant, both in the presence or absence of GlcN (Figure 5). We conclude that Slt2 is an essential effector of TORC1 activity in tunicamycin-exposed cells.

### 3.6. The Proteasome Homeostasis Is Not Critical to Tunicamycin Survival

Downstream TORC1, Slt2 has been reported to control Adc17 and proteasome abundance [68]. Adc17, a stress-inducible RAC (regulatory particle assembly chaperone), is crucial for proteasome assembly and to maintain proteasome levels [71]. Thus, the lack of induction of Adc17 in *slt2* mutant cells has been claimed to be the main determinant of the tunicamycin-sensitive phenotype of the MAPK mutant [68]. To confirm this idea, we first tested the phenotype of cells lacking Adc17. Previous work has reported that the *adc17* mutant is tunicamycin sensitive, although the phenotype was weak and visible by drop test only under extremely high (5 μg/mL) tunicamycin concentrations [68]. As shown in Figure S5, *adc17* mutant cells grew as well as the wild type in the presence of 0.5 μg/mL of tunicamycin, a drug dose that fully inhibits the growth of *slt2*. Likewise, no apparent effect on tunicamycin sensitivity was observed by knockdown of different RACs genes such as *NAS6*, *HSM3*, *RPN4*, and *RPN14* (Figure S5).

**Figure 6:**
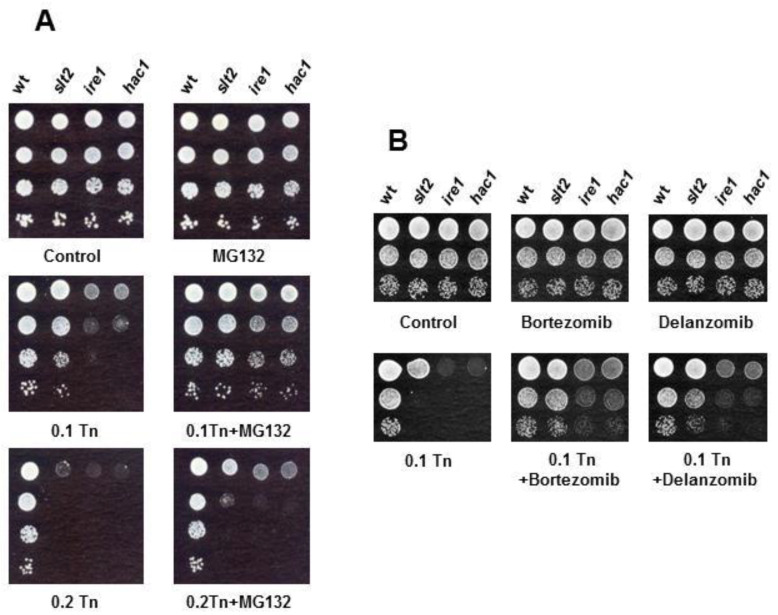
Effects of proteasome inhibitors on tunicamycin tolerance. (**A**) Cultures of the indicated strains, BY4741 wild-type (wt) *slt2*, *ire1*, and *hac1* were assayed for growth on MPD-SDS (0.003%) plates lacking (control) or containing tunicamycin (Tn) at the indicated concentration (μg/mL) and/or 75 μM MG132. (**B**) The same strains that in (**A**) were tested for growth in the presence of tunicamycin (Tn) at the indicated concentration (μg/mL) and/or 50 μM of bortezomib or delanzomib. A representative experiment is shown.

Then, we analyzed tunicamycin sensitivity in the presence of proteasome inhibitors. Previous work by Denzel et al. [30] reported that gfat-1 gain-of-function mutants of *C. elegans* display enhanced proteasome activity. Thus, we reasoned that impaired proteasome activity would cause increased tunicamycin sensitivity. We first tested the effect of MG132, short peptide aldehydes that block active sites of the proteasome [72]. The use of proteasome inhibitors in wild-type *S. cerevisiae* cells is hampered by the impermeability of the cell wall or membrane [73], an issue that can be overcome by the use of a synthetic medium containing L-proline and SDS [74]. As it is shown, proteasome inhibition by MG132 did not result in increased toxicity of tunicamycin in either of the strains analyzed, wild type, *slt2*, *ire1*, or *hac1* (Figure 6A). On the contrary, the inhibitor caused a slight improvement in growth at low doses of tunicamycin, a subtle effect that could be explained by the activation of compensatory mechanisms. Evidence indicates that proteasome inhibition or impairment activates autophagy [75]. Consistent with this, similar results (Figure 6B) were obtained by using bortezomib (also named PS-341), a reversible inhibitor of the proteasome containing a peptide-like backbone and boronate group, or its structurally related inhibitor delanzomib [76].

### 3.7. Knock-Out of SLT2 and Glucosamine Treatment Has Distinct Effects on the Tunicamycin-Induced Autophagic Response

**Figure 7:**
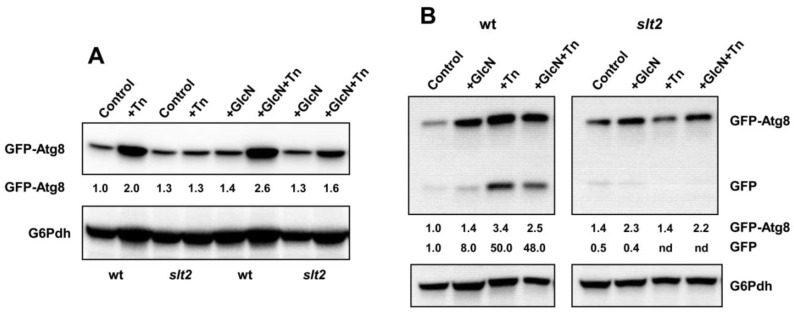
HBP activation and *SLT2* knock-out causes distinct responses to autophagy. (**A**) Overnight SCD-Leu grown cultures of pRS415-GFP-ATG8 transformants of the BY4741 wild-type (wt) and *slt2* strains were refreshed in YPD (OD_600_ = 0.1) lacking or containing 11.5 mM glucosamine (GlcN) and grown at 30 °C until OD_600_ ~ 0.3. Aliquots were withdrawn for their immediate analysis (Control), and cultures were split in two and incubated at 30 °C in the presence (Tn; GlcN+Tn) or absence (control; GlcN) of 2 μg/mL tunicamycin (Tn) for 3 h. Protein extracts were prepared as described in Section 2, separated by SDS-PAGE, and analyzed by Western blot for GFP-Atg8 and free GFP using anti-GFP antibody. The image shows only a part of the gel where GFP-Atg8 was localized as free GFP was hardly detected. (**B**) The same strains were assayed as above except that the tunicamycin (Tn) treatment was extended for 6 h. In all cases, the bands corresponding with GFP-Atg8 or free GFP (GFP) are indicated. The level of glucose-6-phosphate dehydrogenase (G6Pdh) was used as a loading control for crude extracts. The values at the bottom of the images represent the GFP-Atg8 and free GFP abundance relative to that of the wild-type strain under control conditions that was set at 1.0. Representative experiments are shown.

Beyond proteasome regulation, TORC1 suppresses autophagy [64], a self-degradation mechanism that improves proteostasis through the clearance of aggregated proteins [77]. Autophagy induction is triggered by TORC1 inhibition in response to either nutrient starvation or stress conditions, including ER stress [78]. Hence, we tested the effect of HBP activation on autophagy induction, as measured by the GFP-Atg8 processing assay [79]. Wild-type and *slt2* cells were transformed with a plasmid encoding GFP-Atg8 [80] and the abundance of GFP-Atg8 and free GFP after 3 and 6 h in the presence or absence of GlcN and/or tunicamycin was analyzed by Western blot. The ubiquitin-like protein Atg8 is one of the major Atg proteins that is involved in autophagosome expansion [81], and accordingly, *ATG8* is up-regulated following the induction of autophagy at the transcriptional and translational level [79,82]. Finally, the appearance of free GFP monitors the autophagic flux, as Atg8 is rapidly degraded in the vacuole but GFP is not [83]. As expected, we observed increased levels of GFP-Atg8 in tunicamycin-exposed wild-type cells, an effect that was more pronounced after 6 h of treatment (Figure 7). We also noted that the level of GFP-Atg8 was insensitive to tunicamycin treatment in the *slt2* mutant strain (Figure 7), in good correspondence with the absence of TORC1 inhibition in this strain. Interestingly, glucosamine treatment also increased slightly the abundance of GFP-Atg8 in both wild-type and *slt2* cells after 6 h of the onset of the experiment (Figure 7B), suggesting that enhanced activity of the HBP stimulates the autophagy, a result previously reported in *C. elegans* [30].

With regard to the autophagic flux, a band corresponding with free GFP was only observed after 6 h of tunicamycin exposure of wild-type cells (Figure 7B). Likewise, the presence of GlcN appeared to increase the proteolysis of GFP-Atg8 expressed in the wild-type strain, but the effect was weak. Finally, the tunicamycin treatment did not cause the proteolysis of GFP-Atg8 in the *slt2* mutant (Figure 7B). We conclude that Slt2 is required to trigger autophagy in response to ER stress. The activity of the HBP also appears to play a role in stimulating this protective mechanism.

### 3.8. The Bioenergetics Response of Yeast Cells to ER Stress Depends on a Functional Slt2 MAPK

**Figure 8:**
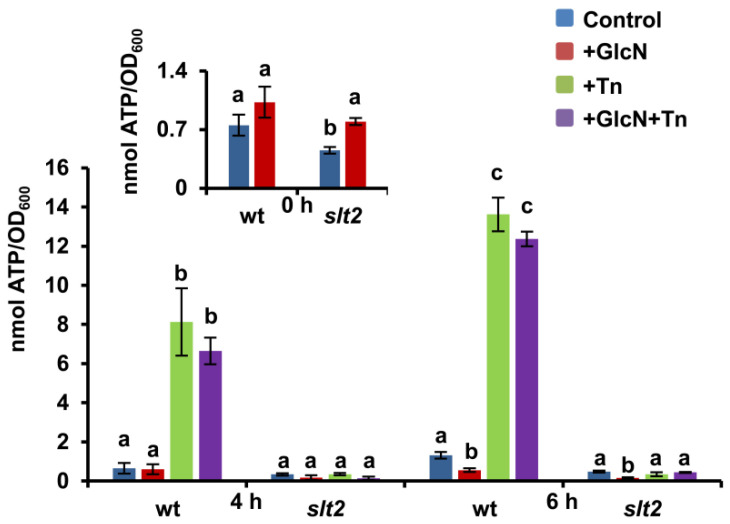
The tunicamycin-induced increase in ATP levels is absent in *slt2* mutant cells. YPD-grown overnight seed cultures of the BY4741 wild-type and *slt2* mutant strain were refreshed at OD_600_ = 0.1 in the same medium lacking or containing 11.5 mM glucosamine (GlcN) and grown at 30 °C until OD_600_ = 0.3. Aliquots were withdrawn for their immediate analysis (time 0), and cultures were split in two and incubated at 30 °C in the presence (Tn; GlcN+Tn) or absence (Control; GlcN) of 2 μg/mL tunicamycin (Tn) for 4 and 6 h. Data are expressed as nmol of ATP per OD_600_ and represent the mean (±SD) of triplicate assays. ATP kinetics for each strain and condition was repeated at least three times. Different letters represent significant differences at *p* < 0.05 probability level for each strain and growth condition compared with the corresponding control.

Previous work indicated that ER-to-mitochondria Ca^2+^ transfer increases during the early phase of tunicamycin exposure to stimulate mitochondrial bioenergetics [84]. As a result, ATP levels, oxygen consumption, and reductive power increase in order to face the energy demand for protein folding and clearance of protein aggregates under ER stress [85]. Evidence also suggests that TORC1 is a central signaling effector of this response as its inhibition by rapamycin mimics the bioenergetics effects of tunicamycin [86]. Therefore, we decided to assess ATP levels in tunicamycin-treated cells of the wild-type and *slt2* strain in the presence or absence of GlcN. As shown in Figure 8, the level of ATP at the onset of the experiment (0 h) was slightly higher in GlcN-containing YPD-grown cells of both, wild-type and *slt2* mutant strain, a result that could be explained in light of the reduced abundance of glycoproteins in these cells (Figure 4). Protein translation is one of the energetically most expensive processes [87]. As expected, the level of ATP increased in wild-type cells after 4 and 6 h of tunicamycin treatment, but not in cells lacking the MAPK Slt2 (Figure 8). We also observed no significant differences by the combined exposure to both tunicamycin and GlcN, indicating that the aminosugar does not interfere, at least at the doses used, with the ATP overproduction upon ER stress.

## 4. Discussion

The idea that the role of Slt2 in the ER-stress protective response was connected to its function as a transcriptional activator of *GFA1* ([24]; this work), came from the finding that increased biosynthesis of UDP-GlcNAc in *C. elegans* improves protein homeostasis [30]. Slt2, the MAPK of the CWI pathway, has been reported to play an important role in ER-stress tolerance, although the exact mechanism is unclear as *slt2* mutant cells show only a weak defect in the activation of the UPR ([18]; this work), the transcriptional program addressed to mitigate the accumulation of unfolded proteins [13,14,15]. Here, we demonstrated that ectopic expression of *GFA1* or supplementation of the growth medium with the aminosugar GlcN confers increased tolerance to different ER-stress inducers and rescues the ER-stress growth defect of *slt2* mutant cells. GlcN is converted by the action of hexokinase to GlcN-6-phosphate [49], which increases the level of HBP intermediates and relieves the need for Gfa1 [50]. Quite remarkably, we also found that activation of the HBP by GlcN addition alleviated the tunicamycin-sensitivity phenotype of cells lacking Hog1, the MAPK of the osmolarity HOG pathway [51], Ire1 or Hac1. Like Slt2, strains lacking Hog1 display sensitivity to tunicamycin with no or minor effects on the UPR-mediated regulation [39]. On the contrary, Ire1 and Hac1 are essential effectors of the UPR and the lack of any of them strongly impairs the transcriptional upregulation of hundreds of genes in response to ER stress [16,44,45]. However, all of them have in common a role in the transcriptional activation of *GFA1* [18,26]. Altogether, our results stress the importance of the hexosamine pathway in the ER-stress protective response in *S. cerevisiae* and the role of Gfa1 as a central effector, whose activity is coordinately controlled by a number of conserved signaling pathways.

Regulating protein degradation is an integral part of the UPR program to relieve ER stress [15,88]. Consistent with this, enhanced proteolysis involving ER-associated degradation (ERAD), proteasomal activity, and autophagy was observed in *C. elegans* in response to increased flux through the HBP [30]. Similarly, HBP activation was found to reduce aggregated polyQ and toxicity in tissue cultures [89]. Although the mechanism involved in these effects was not clarified, it was suggested that enhanced HBP would generate a weak ER stress that, in turn, would increase autophagy through eIF2α phosphorylation [89], the α subunit of the eukaryotic initiation factor-2 [90]. Previous work reported that glucosamine treatment can lead to ER stress [91] and eIF2α phosphorylation [92], whereby reducing polyQ [93] and SDS-insoluble Huntingtin aggregates [94] in animal models.

Unlike this view, we found evidence that is against the idea that GlcN causes ER stress and that increased autophagic activity is on the basis of the role of the HBP in the ER stress response. (1) The addition of GlcN to the culture medium reduced the tunicamycin-induced UPR response and the transcriptional activation of *GFA1* and *KAR2*, the gene encoding the best-known ER chaperone, which is indispensable when facing ER stress [95]. Furthermore, overexpression of *GFA1* or GlcN supplementation leads to a general growth improvement in yeast cells exposed to ER stress. We conclude that in *S. cerevisiae* the aminosugar provides protection against ER stress, instead of being an ER-stress inducer; (2) autophagy induction by GlcN supplementation was weak and the increase in autophagic flux was hardly visible. Neither deletion of *ATG* genes essential for bulk autophagy, such as *ATG1*, *ATG8*, *ATG12*, *ATG13*, or *ATG33*, resulted in increased sensitivity to tunicamycin (Figure S6). Moreover, we did not find changes by GlcN supplementation in the degradation kinetics of Gas1 induced by cycloheximide treatment, suggesting that the aminosugar does not stimulate protein degradation. (3) We observed a global downregulation in the abundance of concanavalin A-labeled N-glycosylated proteins in cells treated with GlcN, an effect that was not observed in previous studies in *C. elegans* [30,89], and that was confirmed by Western blot analysis of CPY and Gas1, two well-known RE-client proteins [60,61,62]; and (4) no differences in the abundance of several glycolytic proteins were observed when GlcN-treated and -untreated cells were compared, suggesting that the downregulation of N-glycosylated proteins abundance was specific and not due to the activation of global proteolysis mechanisms.

The finding that activated HBP by GlcN supplementation causes a decrease in the abundance of N-glycosylated ER-client proteins was in some way surprising as enhanced flux through the HBP increases the content of UDP-GlcNAc that serves as a precursor for N-glycosylation. Nevertheless, different evidence suggests that this observation could be the result of energetic adaptations. Effective glycosylation and folding of proteins require both biosynthetic precursors and ATP. In mammalian cells, numerous surface proteins and growth factors are N-glycosylated, and the extent of this modification is feedback regulated by glucose availability [96,97], which ensures that cells do not engage in anabolic metabolism when nutrients are limiting [98]. An energetic checkpoint that only allows effective receptor glycosylation and folding when ATP is in excess has also been identified [99]. However, the HBP is a non-energy-generating pathway that consumes glucose [100]. Indeed, AMPK, the major energy-sensing effector, the human homolog of yeast Snf1, has been reported to inhibit by phosphorylation GFAT1 [101] in order to reduce the HBP flux when ATP becomes scarce. Based on this, the energy consumed by the HBP activity in cells overexpressing *GFA1* or by GlcN supplementation could be much higher than in optimal growth conditions. Indeed, GlcN treatment rapidly and transiently lowers ATP levels [100] as the aminosugar acts as a glucose analog that is phosphorylated by hexokinase [49]. In addition, GlcN causes transcriptional reprogramming [102] and represses the respiration rate (QO_2_), even more rapidly than glucose [103], which contributes to reducing energy supply. Consistent with all of this, GlcN has been reported to increase the life span of *C. elegans* and aging mice by mimicking a low-carbohydrate diet [104] and it has been proposed as a promising candidate for pharmacological caloric restriction mimetics [105]. Thus, the results of our study showing that the ATP balance in GlcN-treated and -untreated control cells is rather similar suggests that compensatory mechanisms operate to reduce the demand of ATP when the HBP flux is overloaded, which is consistent with a downregulation of N-glycosylated proteins after GlcN addition to the culture medium. How increased HBP activity causes a reduction in ER-client proteins remains unknown. Emerging evidence indicates that ERAD, the principal quality-control mechanism, not only mediates the elimination of structurally abnormal proteins in the ER but also contributes to the regulation of native proteins [106]. More work is required to clarify the GlcN effects on N-glycosylation and if these are functionally linked to ERAD or other quality control of protein folding mechanisms.

ER stress induced by tunicamycin exposure inhibited TORC1 signaling and activated the autophagy and mitochondrial bioenergetics in wild-type cells of *S. cerevisiae*. Indeed, TORC1 inhibition has been reported to be involved [86] in stimulating mitochondrial uptake of Ca^2+^ release by tunicamycin exposure, thereby increasing respiration and ATP production [85], in order to face the enhanced energy requirements under ER stress conditions [84]. A prominent role of TORC1 inhibition in enhancing overall protein degradation by the ubiquitin–proteasome system and autophagy has also been widely established [64,65,66,68]. In particular, inhibition of TORC1 by ER stress was found to induce Adc17 and to increase proteasome abundance in yeast [68]. In our work, the lack of Adc17 and other proteasome subunits did not have noticeable effects on tunicamycin growth. Neither the absence of important *ATG* genes essential for bulk autophagy had apparent consequences on ER-stress sensitivity, at least at the doses of tunicamycin tested in our study. Likewise, autophagy mutants do not appreciably compromise cell survival or genome integrity in genotoxic stress conditions [65]. Nevertheless, these results should be taken with caution as impairment of a protein degradation mechanism may lead to increased activity of alternative systems. Indeed, recent data indicate the presence of connections and reciprocal regulation mechanisms between autophagy and the ubiquitin–proteasome system [107].

**Figure 9:**
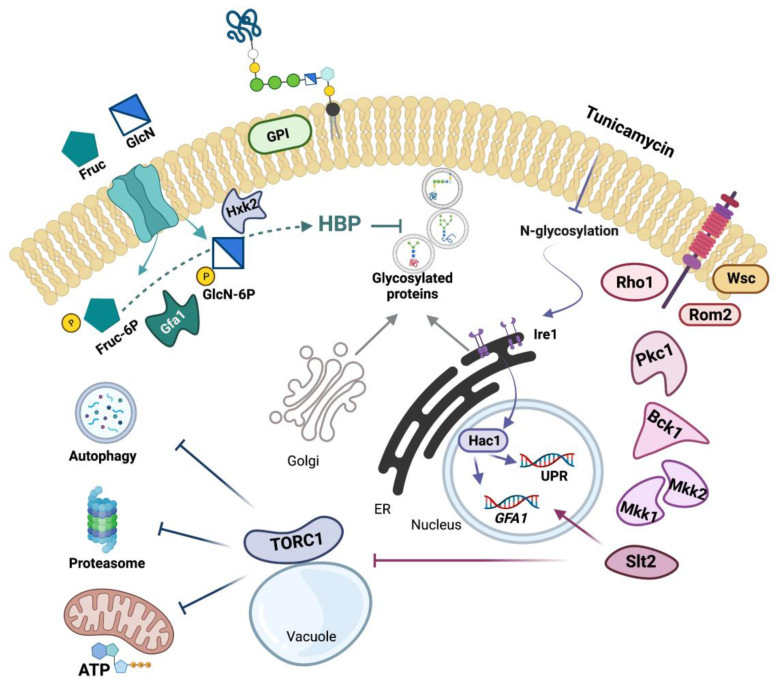
Schematic representation of the ER-stress signaling network and its interaction with Slt2 and the hexosamine pathway. The CWI signaling pathway from the anchored Wsc sensors to the final MAPK Slt2 (see [3,5,6,7,8,9,10] as representative reviews) and the metabolic steps from fructose (Fruc) or glucosamine (GlcN) to glucosamine-6-phosphate (GlcN-6P) catalyzed by Gfa1 [22] and Hxk2 [49,50], respectively, are shown. Gfa1 catalyzes the first committed and rate-limiting step of the HBP, which provides UDP-N-acetylglucosamine for, among others, glycosylation and GPI-anchoring of proteins [20,21]. Tunicamycin exposure induces ER stress by inhibiting the N-glycosylation of proteins [42], which triggers a protective response, the UPR, a transcription program mediated by Ire1 and Hac1, that upregulates the transcription of hundreds of genes [13,14,15,16], among them *GFA1* [24]. Expression of *GFA1* also depends on Slt2 [26], which link the CWI and the UPR in providing increased flux through the HBP under ER-stress conditions, a response that reduces the abundance of ER-client proteins. ER-stress also inhibits TORC1 [66,67], a stress and growth controller [63], which downregulates protein homeostasis mediated by autophagy [64,78] and proteasome [68] activities and the bioenergetics response [84,86], which provides the energy required for protein folding and clearance of protein aggregates under ER stress [85]. Remarkably, Slt2 is required to both *GFA1* expression and TORC1 inhibition in response to ER-stress, which accounts for the strong growth defect of cells devoted of Slt2 in media containing ER-stress-inducers, a phenotype that can be relieved by *GFA1* overexpresion or GlcN supplementation. Arrows and bars denote positive and negative interactions, respectively. For additional details, see the text.

Remarkably, we found that autophagy or enhanced production of ATP was absent in cells lacking Slt2. The most likely explanation is the impaired inhibition of TORC1 associated with the *SLT2* mutation under ER stress. Recently, the role of Slt2 in regulating rapamycin-induced autophagy and TORC1 inactivation has been ruled out [65]. Neither TORC1 inactivation after DNA damage was found to be dependent on Slt2, although autophagy induction was partially reduced by the loss of MAPK [65]. Hence, TORC1 inactivation under ER stress appears to differ mechanistically with respect to other stressful conditions in the requirement of Slt2. Interestingly, previous work has placed Slt2 downstream of TORC1 inhibition in response to caffeine [108] and rapamycin treatment [36], which would mediate the PKA regulation by TORC1 [109]. Rather, our work places Slt2 upstream of TORC1 signaling, although a direct interaction between them seems unlikely. Crosstalk between CWI, PKA, Snf1, and TORC1 signaling has been extensively documented [110] and these are obviously potential effectors in the ER-stress signaling network. Nonetheless, as we graphically summarize in Figure 9, our study highlights the importance of Slt2 in controlling the adaptive response to ER stress by HBP-dependent and -independent mechanisms. Indeed, Slt2 is required both to induce *GFA1* transcription and to inhibit TORC1 in response to ER-stress, which accounts for the strong defect of *slt2* mutant cells under these conditions. This phenotype can be alleviated by hyperactivation of the HBP via *GFA1* overexpression or GlcN supplementation, which reduces the load of ER-incoming proteins and the ER stress.

## Data Availability

Not applicable.

## Supplementary Materials

The following supporting information can be downloaded at: https://www.mdpi.com/article/10.3390/jof8020092/s1, Figure S1: Overexpression of *GFA1* provides enhanced ER stress tolerance; Table S1: *Saccharomyces cerevisiae* strains used in this study; Figure S2: Glucosamine supplementation stimulates the growth of *Saccharomyces cerevisiae* in the presence of the ER-stress inducer tunicamycin; Table S2: Oligonucleotides used in this study; Figure S3: Glucosamine addition partially overcomes the inositol requirement of CWI pathway mutants; Table S3: Plasmids used in this study; Figure S4: Glucosamine reduces Gas1 abundance; Figure S5: Knock-out of RACs does not cause increased ER-stress sensitivity; Figure S6: *ATG* genes essential for bulk autophagy are dispensable for ER stress tolerance.
