# Supplementary material for: Slt2 Is Required to Activate ER-Stress-Protective Mechanisms through TORC1 Inhibition and Hexosamine Pathway Activation
Source: J Fungi (Basel). 2022 Jan 18;8(2):92. doi: 10.3390/jof8020092 (PMC8877190; doi:10.3390/jof8020092)
Supplement: Supplementary file 1 [file jof-08-00092-s001.zip › jof-1531616-supplementary.pdf]

Slt2 is required to activate ER-stress protective mechanisms through TORC1 inhibition and hexosamine pathway activation

Isabel E. Sánchez-Adriá[a], Gemma Sanmartín[a], Jose A. Prieto[a], Francisco Estruch[b], and Francisca Randez-Gil[a],*

[a] Department of Biotechnology, Instituto de Agroquímica y Tecnología de los Alimentos, Consejo Superior de Investigaciones Científicas, Avda. Agustín Escardino, 7. 46980-Paterna, Valencia, Spain

[b] Departament of Biochemistry and Molecular Biology, Universitat de València, Dr. Moliner 50, 46100-Burjassot, Spain

Contents:

Table S1. *Saccharomyces cerevisiae* strains used in this study.
Table S2. Oligonucleotides used in this study.
Table S3. Plasmids used in this study.

Figure S1. Overexpression of *GFA1* provides enhanced ER stress tolerance.
Figure S2. Glucosamine supplementation stimulates the growth of *Saccharomyces cerevisiae* in the presence of the ER-stress inducer tunicamycin.
Figure S3. Glucosamine addition partially overcomes the inositol requirement of CWI pathway mutants.
Figure S4. Glucosamine reduces Gas1 abundance.
Figure S5. Knock-out of RACs does not cause increased ER-stress sensitivity.
Figure S6. *ATG* genes essential for bulk autophagy are dispensable for ER stress tolerance.

Table S1. *Saccharomyces cerevisiae* strains used in this study.

Strain	Genotype	Reference or source
BY4741 (wt)	*MATa ura3Δ his3-Δ1 leu-2Δ met15Δ*	Euroscarf
slt2	BY4741 *slt2::kanMX4*	Euroscarf
Gfa1-TAP	BY4741 *GFA1-TAP::HIS3MX6*	Horizon Discovery
ire1	BY4741 *ire1::kanMX4*	Euroscarf
hac1	BY4741 *hac1::kanMX4*	Euroscarf
chs3	BY4741 *chs3::kanMX4*	Euroscarf
slt2 chs3	BY4741 *slt2::kanMX4 chs3::hphMX4*	This study
reg1	BY4741 *reg1::kanMX4*	Euroscarf
snf1	BY4741 *snf1::kanMX4*	Euroscarf
reg1 snf1	BY4741 *reg1::kanMX4 snf1::natMX4*	[57]
bck1	BY4741 *bck1::kanMX4*	Euroscarf
adc17	BY4741 *adc17::kanMX4*	Euroscarf
rpn4	BY4741 *rpn4::kanMX4*	Euroscarf
hsm3	BY4741 *hsm3::kanMX4*	Euroscarf
nas6	BY4741 *nas6::kanMX4*	Euroscarf
rpn14	BY4741 *rpn14::kanMX4*	Euroscarf
atg8	BY4741 *atg8::kanMX4*	Euroscarf
atg1	BY4741 *atg1::kanMX4*	Euroscarf
atg12	BY4741 *atg12::kanMX4*	Euroscarf
atg13	BY4741 *atg13::kanMX4*	Euroscarf
atg33	BY4741 *atg33::kanMX4*	Euroscarf

Table S2. Oligonucleotides used in this study.

Name	Sequence	Used for
KAN-S2	GTCAAGGAGGGTATTCTGG	Verification integration
CHS3-S1	CATCAAGTACCGGTGTAAACCCAAATGCAACTCGTCGGAGCGTACGCTGCAGGTCGAC	Deletion of *CHS3*. Forward primer
CHS3-S2	GTACTCAAACACTTGAATTGCAGTTACCCAAGATTGTGCCATCGATGAATTCGAGCTC	Deletion of *CHS3*. Reverse primer
CHS3-V1	TTGTAGTAAATCCTGCTAG	Verification deletion *CHS3*
Oligo-dT	TTTTTTTTTTTTTTTTTT(A,C or G)	cDNA synthesis
GFA1-F1	GGTGTTTTGGCAGGTGAGTT	Quantification by qRT-PCR of *GFA1* mRNA. Forward primer
GFA1-R1	GGTTTGCAGGTCGATTGATT	Quantification by qRT-PCR of *GFA1* mRNA. Reverse primer
ACT1-F1	GGATCTTCTACTACATCAGC	Quantification by qRT-PCR of *ACT1* mRNA. Forward primer
ACT1-R1	CACATACCAGAACCGTTATC	Quantification by qRT-PCR of *ACT1* mRNA. Reverse primer

Table S3. Plasmids used in this study.

Plasmid	Description	Source or reference
pMCZ-Y	A high copy 2 μ *URA3* plasmid containing a *UPRE-lacZ* reporter (a gift from Prof Mori).	[43]
pAG32	The plasmid contains the hph gene from *Klebsiella pneumoniae* encoding hygromycin B phosphotransferase and confers resistance to the antibiotic hygromycin B.	[34]
pRS415-GFP-ATG8	Centromeric plasmid containing a fusion of GFP at the N-terminus of *ATG8.*	[80]
pFCM493	YEp13 (*LEU2*) derivative containing the *GFA1* gene	[48]
pKK1	Centromeric plasmid with *LEU2* selection marker.	[46]
pMS109 (pHAC1)	Fragment containing the *HAC1* gene without introns inserted between the promoter and terminator of *PGK1* in pKK1.	[46]

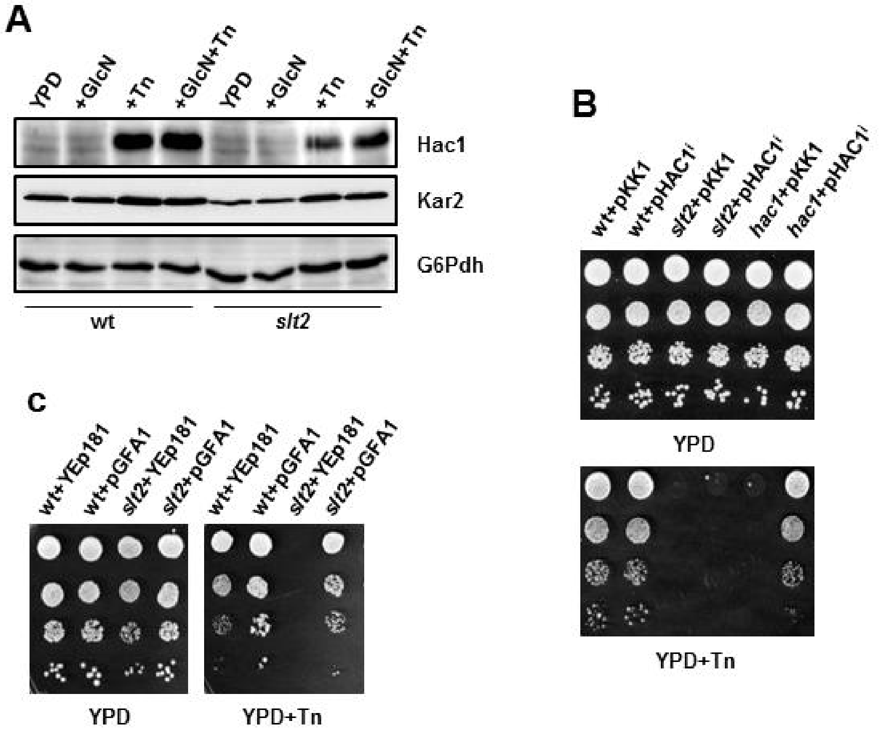

Figure S1. Overexpression of *GFA1* provides enhanced ER stress tolerance. (A) Protein extracts from wild-type (wt) and *slt2* mutant strains were analyzed by SDS-PAGE and Western blot by using Hac1 and Kar2 antibodies as described in the Materials and Methods section. YPD-grown cultures containing or lacking 11.5 mM glucosamine (GlcN) were grown until mid-log-phase (OD_{600} ~ 0.5) at 30 °C (control, YPD) and treated with 2 µg/ml Tn for 90 min. The level of glucose-6-phosphate dehydrogenase (G6Pdh) was used as a loading control for crude extracts. (B) Serial dilutions ($1\text{-}10^{-3}$) of SCD-Ura-grown cultures (OD_{600} ~ 0.8) of pKK1 (control plasmid) and $pHAC1^{i}$ transformants of the BY4741 wild-type (wt), *slt2* and *hac1* mutant strains were spotted (2 µl) onto YPD plates that lacked (YPD) or contained tunicamycin, Tn (0.5 µg/ml; YPD+Tn) and were incubated at 30 ºC for 2-4 days. The plasmid $pHAC1^{i}$ express the mature/spliced and active mRNA of *HAC1*. (C) Drop test of BY4741 wild-type (wt) and *slt2* mutant strains transformed with plasmids YEplac181 (empty plasmid; YEp181) or pFCM493 (pGFA1). Cultures were diluted and spotted on YPD or tunicamycin-containing YPD (YPD+Tn) as described in (B). In all cases, representative experiments are shown.

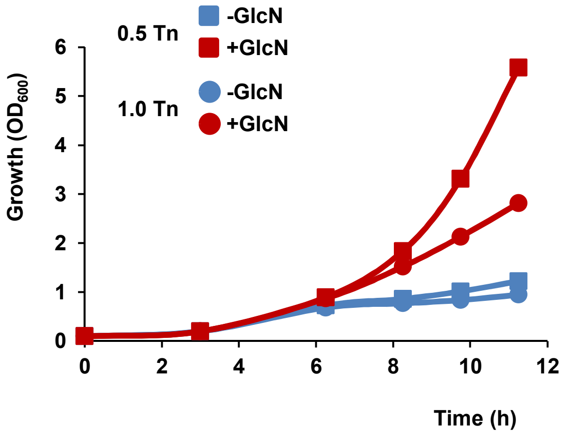

Figure S2. Glucosamine supplementation stimulates the growth of *Saccharomyces cerevisiae* in the presence of the ER-stress inducer tunicamycin. Overnight YPD-grown cultures of the BY4741 wild-type strain were refreshed in YPD (OD_{600} = 0.1) lacking (-GlcN; blue lines and symbols) or containing 11.5 mM glucosamine (+GlcN; red lines and symbols) and grown at 30°C for 3 h. Cultures were split in two and incubated at 30 ºC in the presence of 0.5 µg/ml of tunicamycin (squares) or 1.0 µg/ml of the same drug (circles). Growth was followed by measuring the OD_{600} at the indicated times. A representative experiment is shown.

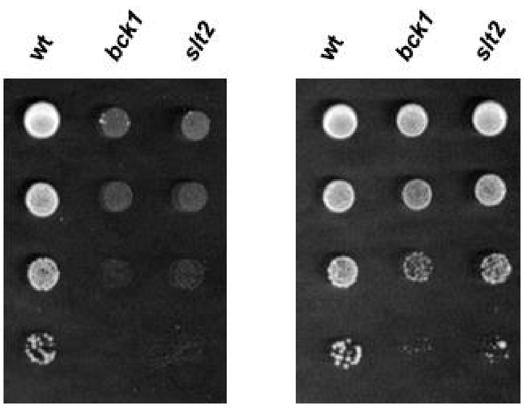

Figure S3. Glucosamine addition partially overcomes the inositol requirement of CWI pathway mutants. YPD-grown cultures (OD_{600} ~ 0.8) of the BY4741 wild-type (wt), *bck1*, and *slt2* mutant strains were centrifuged and the pellets were washed with water, resuspended in SD-INO medium at the same OD_{600}, and serial dilutions ($1\text{-}10^{-3}$) were spotted (2 µl) onto SD-INO plates lacking (SD-INO) or containing 11.5 mM glucosamine (SD-INO+GlcN). Plates were incubated at 30 ºC and inspected for growth after 2-4 days. A representative experiment is shown.

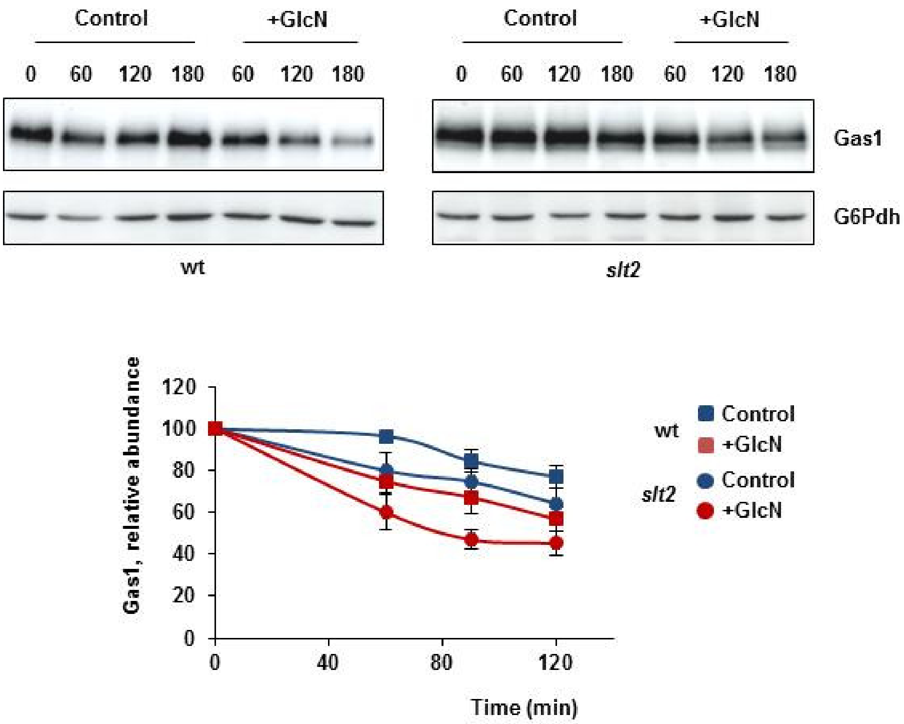

Figure S4. Glucosamine reduces Gas1 cellular content. Cells of the wild-type BY4741 (wt) and *slt2* mutant strains were grown to the mid-logarithmic phase (OD_{600} ~ 0.5) in YPD medium at 30 ºC. An aliquot was withdrawn for the analysis (time 0) and the rest of the culture was treated with 11.5 mM glucosamine (GlcN) for the indicated times. Protein extracts from untreated (time 0; control) and GlcN-treated samples were separated by SDS-PAGE, and Gas1 abundance was analyzed by Western blot and immunodetection by using a rabbit polyclonal anti-Gas1 antibody as described in the Materials and Methods section. The level of glucose-6-phosphate dehydrogenase (G6Pdh) was used as a loading control for crude extracts. The graph shows the abundance of Gas1 in each time point relative to that of the corresponding control (time 0) for untreated and GlcN-treated samples of each strain analyzed. Data are the mean (±SD) of three independent biological replicates.

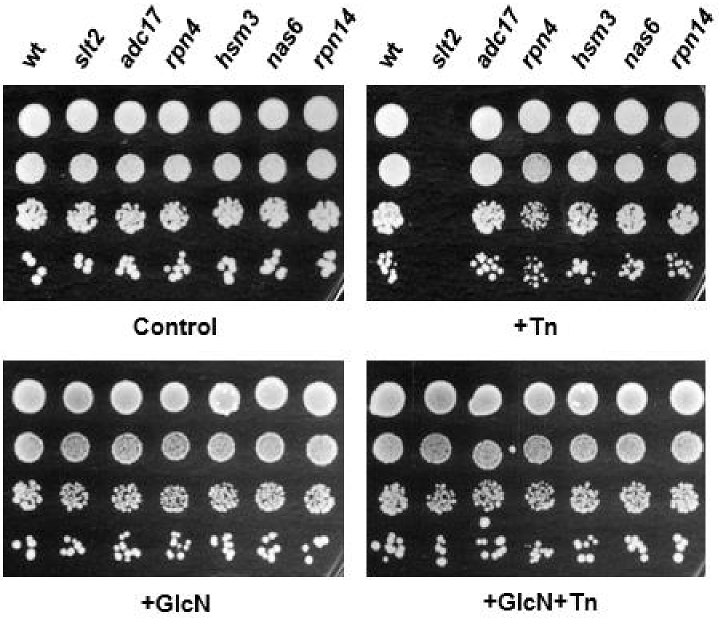

Figure S5. Knock-out of RACs does not cause increased ER-stress sensitivity. Cultures of BY4741 wild-type (wt), *slt2* and stress-inducible RAC (regulatory particle assembly-chaperone) deletion strains lacking *ADC7*, *RPN4*, *HSM3*, *NAS6* or *RPN14* were diluted ($1\text{-}10^{-3}$) and spotted (2 µl) on plates containing or lacking 11.5 mM glucosamine (GlcN) and/or 0.5 µg/ml tunicamycin (Tn). Plates were incubated at 30 ºC and inspected for growth after 2-4 days. A representative experiment is shown.

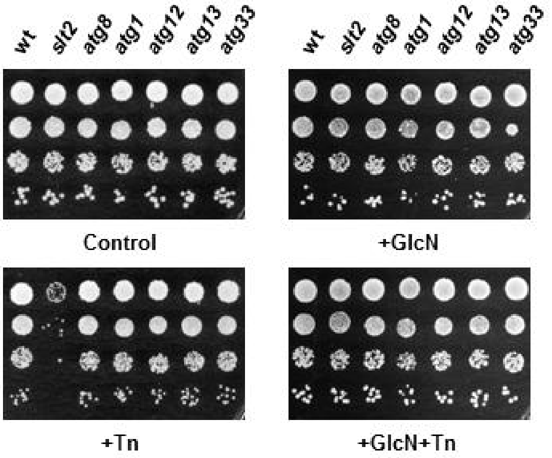

Figure S6. *ATG* genes essential for bulk autophagy are dispensable for ER stress tolerance. Drop test of BY4741 wild-type (wt) and *slt2*, *atg8*, *atg1*, *atg12*, *atg13* and *atg33* mutant strains was carried out on plates lacking or containing 11.5 mM glucosamine (GlcN) and/or 0.5 μg/ml tunicamycin (Tn).
